# Combined scanning probe electronic and thermal characterization of an indium arsenide nanowire

**DOI:** 10.3762/bjnano.9.15

**Published:** 2018-01-11

**Authors:** Tino Wagner, Fabian Menges, Heike Riel, Bernd Gotsmann, Andreas Stemmer

**Affiliations:** 1 ETH Zürich, Nanotechnology Group, Säumerstrasse 4, 8803 Rüschlikon, Switzerland; 2 IBM Research – Zurich, Säumerstrasse 4, 8803 Rüschlikon, Switzerland

**Keywords:** contact resistance, Kelvin probe force microscopy (KFM), nanowire, scanning thermal microscopy (SThM), self-heating

## Abstract

As electronic devices are downsized, physical processes at the interface to electrodes may dominate and limit device performance. A crucial step towards device optimization is being able to separate such contact effects from intrinsic device properties. Likewise, an increased local temperature due to Joule heating at contacts and the formation of hot spots may put limits on device integration. Therefore, being able to observe profiles of both electronic and thermal device properties at the nanoscale is important. Here, we show measurements by scanning thermal and Kelvin probe force microscopy of the same 60 nm diameter indium arsenide nanowire in operation. The observed temperature along the wire is substantially elevated near the contacts and deviates from the bell-shaped temperature profile one would expect from homogeneous heating. Voltage profiles acquired by Kelvin probe force microscopy not only allow us to determine the electrical nanowire conductivity, but also to identify and quantify sizable and non-linear contact resistances at the buried nanowire–electrode interfaces. Complementing these data with thermal measurements, we obtain a device model further permitting separate extraction of the local thermal nanowire and interface conductivities.

## Introduction

Electronic and thermal properties of nanoscale devices are innately coupled. The charge carriers in most conductors release energy by scattering at defects or phonons resulting in Joule heating. Furthermore, thermoelectric effects cause a heat flux and result in additional temperature gradients. With further miniturization of electronic devices, such as transistors in integrated circuits, contact resistances and local transport properties govern device performance on a length scale of only a few nanometres. At the same time, Joule dissipation affects devices as they are scaled down, and the increasing power densities require adequate thermal management to avoid performance degradation or failure [1]. Hot spots can appear due to increased local energy dissipation at material or doping interfaces and at defects or constrictions, but also in areas of strong local thermal insulation. To differenciate between different origins of hot spots from temperature data alone is oftentimes not possible. Nanometre-sized hot spots can strongly influence electrical transport through active devices both in positive and negative ways. In conventional logic devices, hot spots lead to signal degradation and reliability issues. In certain memristive devices, however, the breaking and formation of bonds upon local self-heating is the basis of their function [2].

The importance of electro-thermal effects on nanoscale devices is not matched with an in-depth understanding of the relevant effects and material properties. Not all parameters needed for faithful device modelling are readily available, in particular since many of these are size-dependent quantities. Hence, there is an increasing need for characterization techniques able to map both electronic and thermal properties down to the relevant length scales in operating devices. Unlike optical techniques that are limited by diffraction to a resolution of several hundred nanometres, scanning probe methods rely on the interaction of a nanoscale tip with the surface. So far, electrical and thermal device characterization by scanning probe microscopy has often suffered from low lateral resolution [3–6], long-distance averaging effects [7–9], and topography-induced crosstalk [10–12], allowing for a mainly qualitative data interpretation. Here, we demonstrate high-resolution measurements of an operating indium arsenide (InAs) nanowire (NW) by scanning thermal microscopy (SThM) and Kelvin probe force microscopy (KFM), and how the information obtained by both methods can be combined to extract quantitative thermal and electronic device properties.

SThM relies on the measurement of the heat flux between a heated cantilevered tip and a sample surface [13]. When scanning across the surface, this flux is modulated according to the local thermal resistance and temperature difference. To separate the contributions and obtain temperature fields, it is necessary to find the thermal resistance at each point from a reference scan, or to employ an ac mode of operation. In the latter, the current through the device is modulated, and consequently there is a static and dynamic temperature response, allowing signals to be separated and the temperature to be detected with high sensitivity [12].

KFM is a non-contact scanning probe microscopy technique to measure local electrostatic potentials with high lateral resolution. The electrostatic force induced by an ac voltage bias between tip and surface is minimized by adjusting a dc voltage applied to the tip [14]. The resulting local contact potential difference, *U*_lcpd_, depends on the work functions of tip and surface, voltages applied to tip and sample, and charges trapped inside insulators. In two-terminal devices, the voltage profile induced by a constant current bias can be traced by KFM. The voltage drop at interfaces to electrodes directly translates to the contact resistance, allowing one to separate contact and channel resistances. Thereby contact resistance values can be extracted even from small samples, for which four-probe methods or transmission-line methods cannot be applied reliably.

Other scanning probe methods sensitive to surface electronic properties, for example conductive atomic force microscopy (c-AFM) [15] or scanning tunnelling potentiometry (STP) [16], require a current passing through the tip at each point. As such, the tip–sample contact geometry and loading force heavily affect the measurement. Whereas contact issues can be partly alleviated by measuring current at specific points in a force–distance curve, surfaces passivated by a thin oxide remain inaccessible by these methods. Furthermore, for measurements on active devices, measurements by c-AFM are convoluted and difficult to interpret. Depending on its potential, the conductive probe acts like a local current source or sink, and may thereby disturb device operation.

Conversely, KFM, being a non-contact technique, operates with no dc current flow. Since the detection relies on the long-range electrostatic force between surface structures and the tip, care has to be taken to minimize long-range averaging effects on device structures with exposed electrodes [9]. Highest resolution and accuracy is enabled by detecting the force gradient [17–19]. On typical devices with large topography, geometrical artefacts and feedback problems can be minimized by appropriate control schemes [20].

The typical lateral resolution of our SThM and KFM setups is on the order of the tip radius (below 10 nm), at a noise level of 20 μK·Hz^−0.5^ and 1 mV·Hz^−0.5^, respectively, depending on operating conditions and tip shape [21–23].

## Results and Discussion

**Figure 1:**
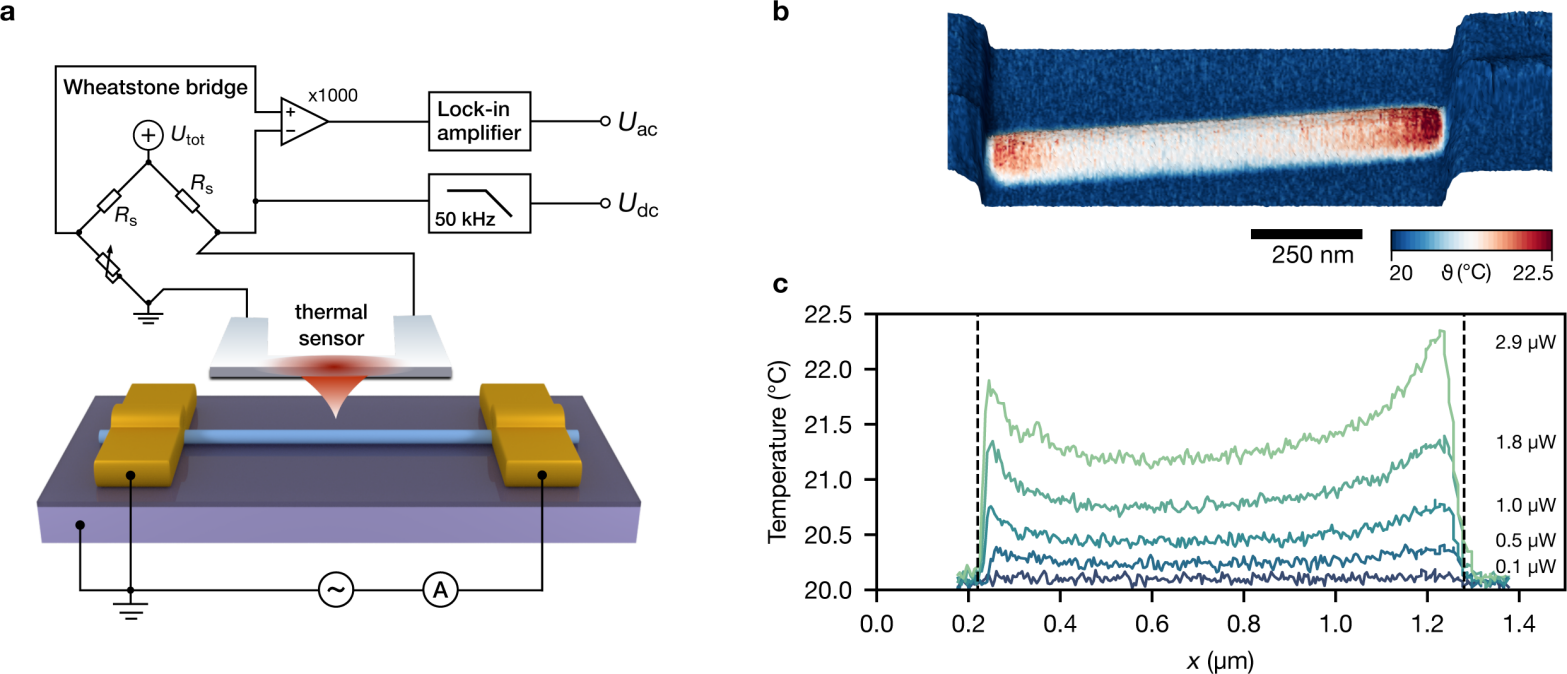
Scanning thermal measurements of the InAs nanowire. (a) Setup for SThM measurements. (b) Topography and superimposed temperature at an average power of 2.9 μW. (c) Temperature profiles along the nanowire at different average power dissipations.

Figure 1a shows the setup for SThM measurements of the InAs nanowire. The wire is driven by a sinusoidal ac voltage bias at 10 kHz, and the two-terminal current is measured using a current amplifier (DHPCA-100, Femto).

The temperature map at an average power of 2.9 μW and a peak current of 30 μA is shown superimposed as a colour code on the topography scan in Figure 1b. From topography, the wire height is 60 nm. On both sides, the wire is electrically contacted by 120 nm high Au/Ni top electrodes, leaving a wire segment of approx. 1 μm in between.

Temperature profiles along the centre of the wire at different average power dissipation are shown in Figure 1c. For a homogenously Joule-heated one-dimensional conductor between two heat-sinking electrodes, the heat diffusion equation predicts a parabolic or bell-shaped temperature profile [24–25]. As seen in our data, the temperature profiles do not follow this trend, but instead show an inverse parabola with increased temperatures towards the electrical contacts. Similar temperature profiles were observed already in previous measurements of a 120 nm diameter InAs nanowire [12]. This behaviour could be caused by either inhomogeneous heat generation (e.g., enhanced Joule heating at the contact regions) or an inhomogeneous thermal coupling between nanowire and heat-sinking substrate. Thermal measurements alone are insufficient to clearly differentiate between the two.

**Figure 2:**
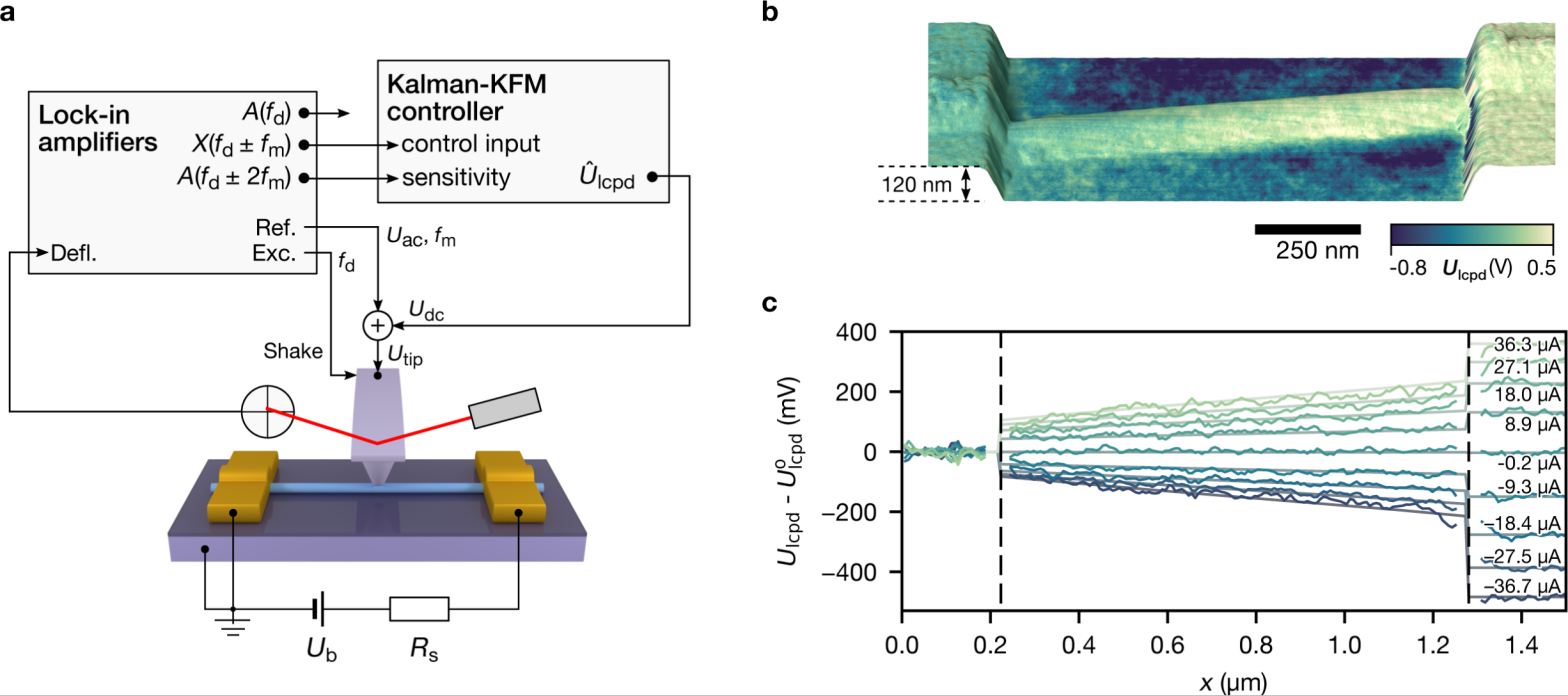
Surface potential measurements of the InAs nanowire. (a) Setup for KFM measurements. (b) Topography and superimposed surface potential at a current of 37.7 μA. (c) Voltage drop along the nanowire at different currents. Surface potential measurements at each bias are corrected by the static voltage offset present in all profiles (see Figure S4 in Supporting Information File 1).

We now turn to the complementary information obtained by KFM. Figure 2a shows our setup for KFM measurements. The nanowire is driven by a dc voltage bias and the two-terminal current is inferred from the voltage drop across a 100 kΩ shunt resistor.

For accurate surface potential measurements and to minimize the effect of long-range electrostatic forces between the cantilever and sample structures, force-gradient sensitive detection is required [7,19]. In our setup, this is assured by direct demodulation of the sidebands that appear upon electrical modulation of the tip–sample electrostatic force [20].

Figure 2b shows the topography and surface potential obtained on the same InAs nanowire as shown in Figure 1. Owing to the particular KFM detection method, we do not observe long-range lateral averaging of the surface potential, and the nanowire and electrodes appear well defined. We recorded surface potential profiles along the centre of the nanowire during consecutive sweeps of the voltage bias. The two-terminal current thereby assumed 50 different values between −38 and +38 μA for each sweep, and several lines were recorded and averaged for each constant bias. The measured surface potential is thus a function of position and current, *U*_lcpd_(*x*, *I*).

Because here we are interested in the effect of current passing through the nanowire, the raw dataset (see Supporting Information File 1, Figure S4) needs to be corrected for the influence of material differences or charges trapped in a passivation layer. For our analysis, we separate a bias-independent offset, 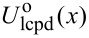, from the raw measurements. A good first-order approximation to this offset is the zero-bias profile, *U*_lcpd_(*x*, *I* = 0) [26]. However, the offset can depend weakly also on bias, since the population and depletion of traps is bias-dependent [27]. Furthermore, random fluctuations of trap potentials over time are not included in a simple baseline subtraction. We account partially for the latter by subtracting instead an average offset present in the complete dataset, which we obtain as a by-product from the fitting method outlined below. The resulting corrected voltage profiles are shown in Figure 2c.

The potential on the grounded left electrode remained zero as the current was swept. Moreover, the surface potential in the oxide-covered region between the electrodes exhibits no appreciable differences when changing the bias (Supporting Information File 1, Figure S3), confirming the reduction of long-range effects due to detection of the force gradient. Being sensitive to the electrostatic force instead, AM-KFM measurements of similar nanowires [4,28–30] have previously shown pronounced lateral averaging, reducing both accuracy and resolution of the measurements. Whereas the nanowire potential is symmetric around 0 μA near the left contact, we observe a pronounced asymmetry at the transition from the nanowire to the electrode on the right hand side. This indicates that the contact resistance here depends on polarity, hinting at the formation of a Schottky barrier between the wire and the electrode.

Along the nanowire, the potential drop behaves mostly linear, indicating uniform transport properties. The potential gradient in the nanowire segment near the electrodes appears slightly increased due to the reduced wire diameter and increased resistance per unit length in this region (Supporting Information File 1, Figure S1 and Figure S2).

Next, we determine a position-dependent resistivity by fitting synthetic data of *U*_lcpd_(*x*, *I*) to the measured dataset. This also enables us to separate contact resistances from the channel behaviour. We assume that at each position *x*, *U*_lcpd_(*x*, *I*) can be separated into an offset value 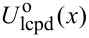 and the potential *U*(*x*, *I*) induced by the applied current *I*. 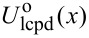 contains information on the effect of charges trapped in a capping oxide surrounding the nanowire, and the differences in work function of the heterogeneous device and the AFM tip. The potential *U*(*x*, *I*) is given by an equivalent resistor model of the nanowire. Because there are top contacts in our device, the nanowire–electrode interfaces are buried, and the potential probed by KFM corresponds only to the voltage of the respective electrode, referred to ground level at the source electrode. The wire region is modelled as a linear chain of resistors *R_i_* = ρ′(*x_i_*)Δ*x* at positions *x_i_* along the nanowire equally spaced by Δ*x*, where ρ′ is the local resistivity ρ normalized by the cross-sectional area *A*. While resistances *R_i_* in the chain may in general depend on the applied current, for example at p–n junctions, we found excellent agreement to a purely ohmic model. This is expected for the homogeneous nanowire in our device. Contact resistances at the source and drain side are modelled using a series of powers in *I*, where we only consider terms up to *I*^2^ to prevent overfitting. To capture diode-like behaviour at the electrical contacts, we consider separate coefficients for positive and negative biases. At zero bias, we force continuity of the contact resistance.

Our reconstruction algorithm minimizes the mean square deviation of measurements and model. To obtain smooth curves of the wire resistivity ρ′(*x*) and to avoid overfitting, we add total variation regularization terms for ρ′ and its gradient. (A complete description of the reconstruction method is given in Supporting Information File 1.)

**Figure 3:**
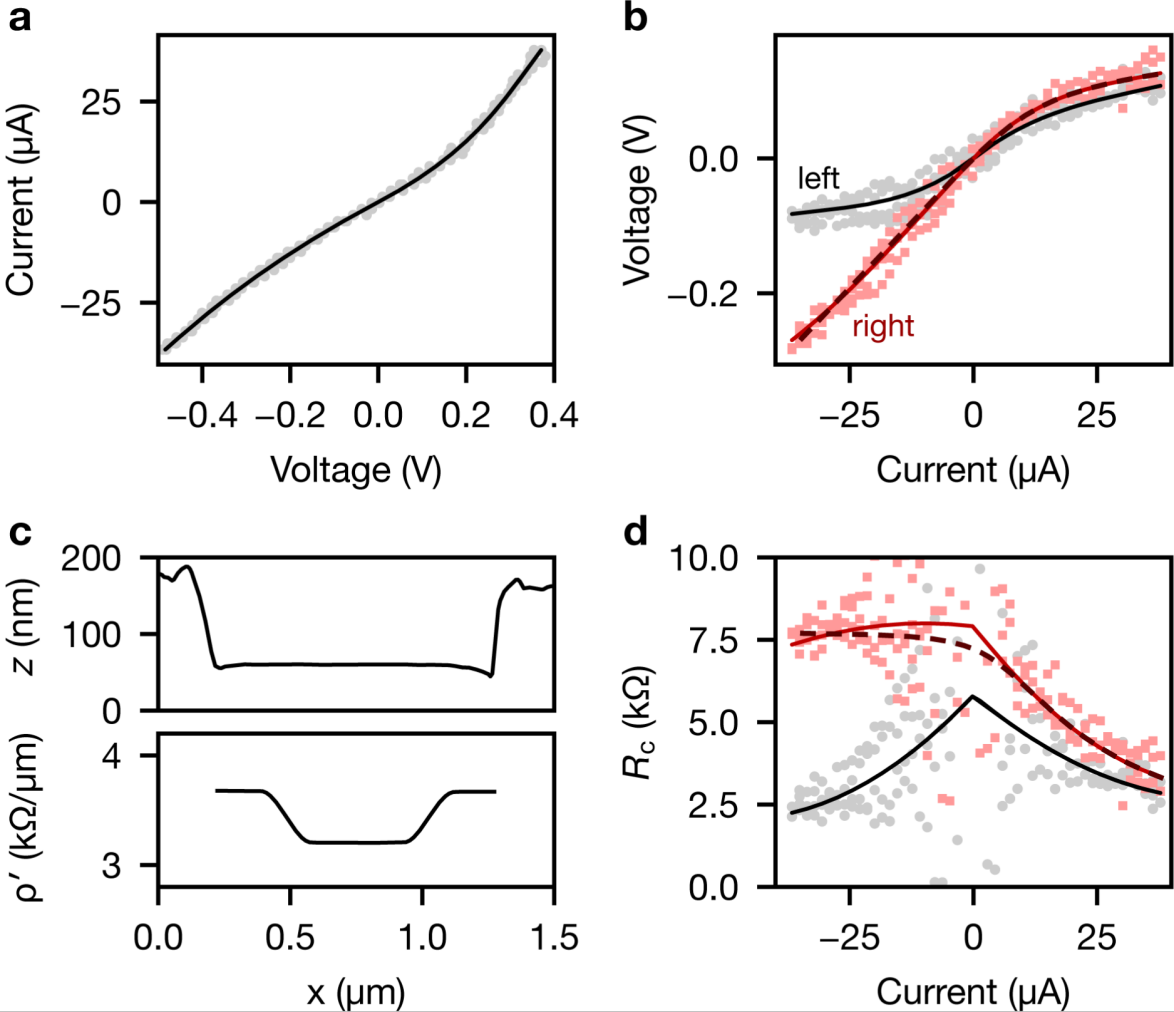
Electrical characteristics extracted from KFM measurements. (a) Two-terminal *I*–*V* characteristics of the nanowire. (b) Voltage drop of the left (black) and right (red) contact as a function of applied current. (c) Profiles of height and reconstructed wire resistivity ρ′. (d) Resistances of the left and right contact. Solid lines are fits from the obtained nanowire model. The scattered points result from a simple analysis of contact resistances from the voltage drop from electrodes to linear fits of the wire region. Dashed lines in (b) and (d) are fits to a leaky diode model for the right contact.

In Figure 3 we show electrical properties of the nanowire extracted through the reconstruction method from sweeps of the bias current in Figure 2b.

Two-terminal current–voltage (*I*–*V*) characteristics of the nanowire device, shown in Figure 3a, are calculated given the voltage drop across the source and drain electrodes, and the known current *I* passed through the nanowire. The solid line is extracted from the reconstructed nanowire model *U*(*x*, *I*), whereas the scattered points are calculated from the raw measurements corrected by the static offset, 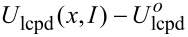. The nanowire device displays mostly linear *I*–*V* characteristics with a slight suppression of the conductance near zero bias. Because in our model we assume ohmic conduction in the wire region, these deviations must originate from behaviour at the contacts.

Figure 3b shows the voltage drop from the left and right contacts to the nanowire as a function of applied current. Again, we show for each current both the voltage drop extracted from the reconstructed model directly (solid line), and values extracted from the offset-corrected raw measurements (markers). In the latter case, we obtain for each current bias a linear fit in the nanowire region between the electrodes. The voltage drop for each electrode is then found by subtracting the average electrode potential from the extrapolated nanowire potential near the electrode edge. The results obtained by both methods are in good agreement. The scattered points provide an easy means to detect potential systematic errors in the model or overfitting. The data does not significantly deviate from the asymmetric quadratic contact model chosen above.

The left contact is nearly symmetric with zero-bias suppression of current due to tunnelling at the interface. The right contact behaves similarly to the left contact for positive bias. For negative current bias, however, compared to the left contact a much larger voltage drop is produced. This indicates the formation of a Schottky diode at the right contact, in which current is suppressed for negative voltage bias. The nearly linear slope for negative bias indicates leakage through interface states. Fitting the contact *I*–*V* behaviour to a leaky diode model (dashed lines in Figure 3b,d; see Supporting Information File 1 for details) results in a parallel resistance of *R*_p_ ≈ 7.8 kΩ, a saturation current of *I*_s_ ≈ 0.28 μA and an ideality factor of η ≈ 1.12 for operation at room temperature, *T* = 300 K.

In Figure 3c, we show the average topography during the line scans and the specific resistivity ρ′ of the nanowire segment obtained from the fit. The resistivity increases from ρ′ = 3.2 kΩ·μm^−1^ in the centre to 3.7 kΩ·μm^−1^ near the electrodes, corresponding to bulk resistivities of approx. 1 mΩ·cm (assuming a circular cross section). Note that the flat segments with smooth transitions are a result of the chosen regularization. Nevertheless, this behaviour captures the increased slopes close to the electrodes already discussed for Figure 2c.

Figure 3d shows contact resistances of both contacts calculated from the data in Figure 3b. The contact resistance at the left electrode reaches approx. 5 kΩ near zero bias, but diminishes to approx. 2.5 kΩ at large current bias. The contact resistance of the right electrode is nearly constant (approx. *R*_p_) for negative bias, but reduces to approx. 2.5 kΩ for large positive currents.

With the contact geometry and assuming a transmission line model for the contact resistances, we also obtained contact resistivities and transfer lengths for both electrodes (Supporting Information, Figure S5). For both electrodes, the contact resistivities are on the order of 1 × 10^6^ Ω·cm^2^ with transfer lengths below 1 μm.

With the KFM measurement and data analysis, we were able to extract all key parameters describing the electronic behaviour of the nanowire device. This model allows us to also predict the thermal behaviour and to compare with SThM measurements, given the thermal conductivity of the nanowire, κ, and thermal couplings to substrate and electrodes.

Since the model obtained by KFM measurements describes the device under dc bias, and SThM was performed under ac bias, a direct comparison is not possible. Therefore, we compare both measurements at the same average power dissipated at nanowire and contacts. For each power in the SThM dataset, we find the bias conditions for an equivalent average power using the model under sinusoidal ac current bias. Then, for these conditions, we obtain the distributed average power 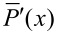 from Joule heating along the nanowire and contact regions. With 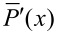 known, we are able to solve the one-dimensional heat equation for the nanowire. κ and thermal couplings to substrate and electrodes are found from a least-squares fit to the SThM measurements in Figure 1. (For details on fit and error estimation, see Supporting Information File 1.)

**Figure 4:**
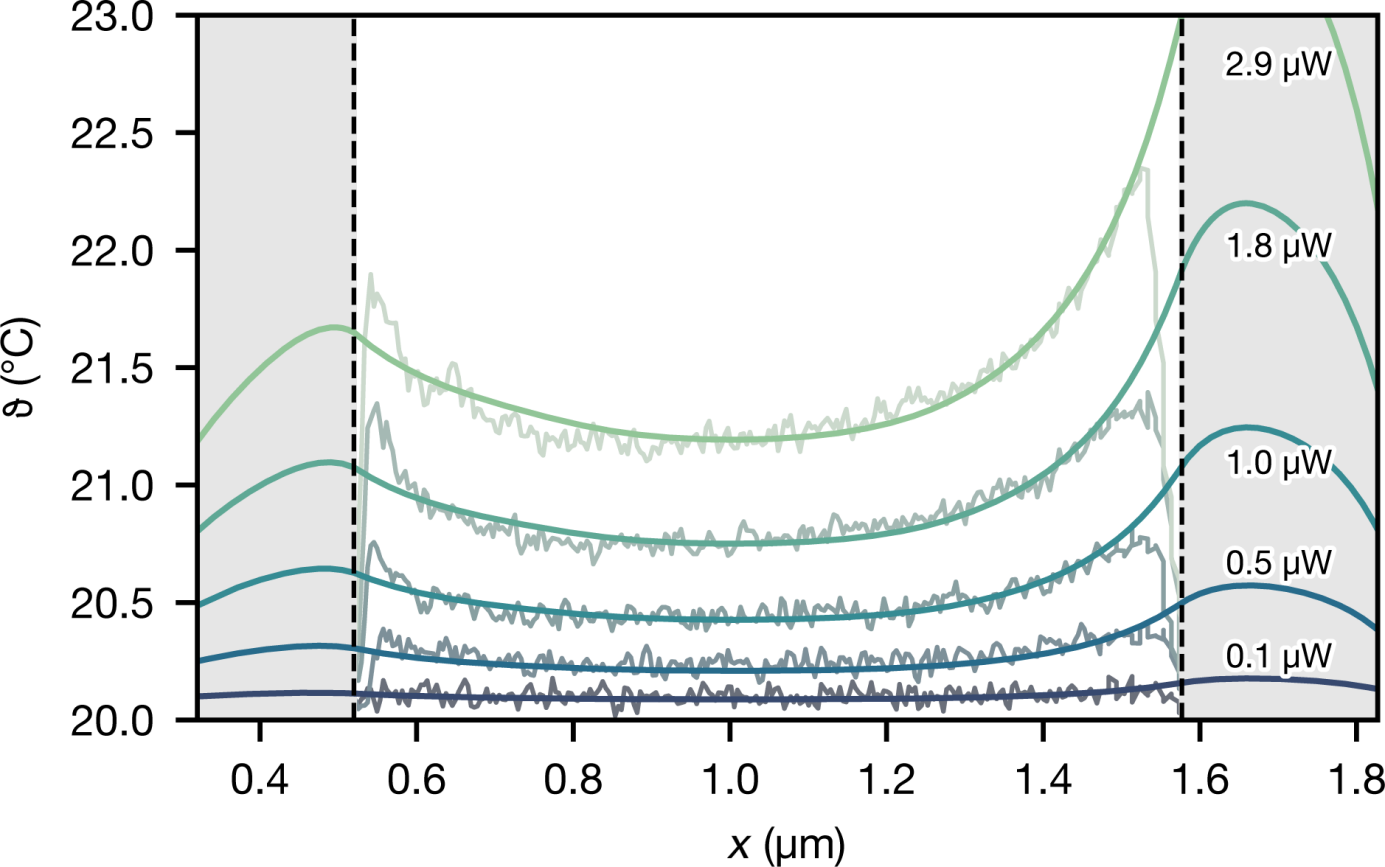
Temperature profiles along the nanowire simulated from its electrical characteristics. The nanowire thermal conductivity (κ = (3.0 ± 1.4) W·m^−1^·K^−1^) and thermal conductances in the substrate (*g*_s_ = (0.6 ± 0.2) W·m^−1^·K^−1^) and electrode regions (*g*_e_ = (1.4 ± 0.4) W·m^−1^·K^−1^) are obtained from a least-squares fit to the scanning thermal measurements. Shaded areas correspond to nanowire sections situated below the top contacts.

Figure 4 shows the fitted temperature profiles together with the SThM data. The inverse-parabolic shape of the profiles is explained by a similarly strong coupling of the nanowire to the substrate, *g*_s_ and in electrode regions, *g*_e_. Joule heat produced at the contacts therefore diffuses into the nanowire regions nearby, causing a rise in temperature near the electrode edges. Likewise, the asymmetry of the temperature measurements is correctly reproduced. Its origin is the different behaviour of both contacts, as seen in Figure 3. (Note that the electrodes, indicated by the gray-shaded areas, render the nanowire temperature beneath them inaccessible to SThM.)

The thermal conductivity of the InAs nanowire, κ = (3.0 ± 1.4) W·m^−1^·K^−1^, is in good agreement with measurements of similar InAs nanowires in microelectromechanical heater/sensor setups [31–32], or with measurements of a 40 nm thick InAs nanofilm [33]. Note that stacking faults perpendicular to the growth direction limit the thermal conductivity of our nanowire [31,34], compared to higher-quality crystalline InAs nanowires [35].

We calculate the interfacial thermal conductivity to the substrate and electrodes along the perimeter of the nanowire from the thermal conductances in the substrate and electrode regions, *g*_s_ and *g*_e_, respectively. Assuming a contact width of half the nanowire diameter for a hexagonal cross section [32], *W*_s_ = *d*_wire_/2, the thermal conductivity in the substrate region is τ_s_ = *g*_s_/*W*_s_ = (2.0 ± 0.6) × 10^7^ W·m^−2^·K^−1^.

In order to obtain the thermal interface conductivity to the metallic contacts, we consider a contact perimeter of half the nanowire circumference, 
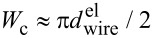
, where we employ a reduced diameter of 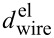 = 50 nm, as estimated by scanning electron microscopy (Supporting Information File 1, Figure S1). Assuming that the interfacial conductance to the substrate, *g*_s_, is the same below electrodes, we obtain 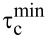 = (*g*_e_ − *g*_s_)/*W*_c_ = (1.0 ± 0.4) × 10^7^ W·m^−2^·K^−1^. This value constitutes only a lower limit, however, since the nanowire is etched below the electrodes. As an upper limit, we consider the interfacial conductivity without heat flow to the substrate, 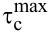 = *g*_e_*W*_c_ = (1.8 ± 0.6) × 10^7^ W·m^−2^·K^−1^. Both values are below the interfacial conductivity of the nanowire to the oxide. This is not unusual, as shown before for GaN nanowires [36]. Because the exact contact geometries are unknown, the error in the estimation of τ_c_ is rather large, and could be reduced considerably by cross-sectional measurements of the contact area.

As shown above for this nanowire (Figure 3), the electrical contacts were partly limited by tunnelling through a thin native oxide layer. If we consider the electron contribution to the thermal conductance to be negligible [36], then phonon scattering at an additional interface may partly be responsible for the reduced interfacial conductivity to the electrodes. The extracted interfacial thermal conductivities agree well with measured values reported in literature [36–39].

## Conclusion

In summary, we have shown that KFM and SThM measurements of the same nanowire device are a powerful combination for a complete electronic and thermal characterization.

KFM alone can be applied to extract key electronic device parameters such as the nanowire resistivity and bias-dependent contact resistivities. Furthermore, when contacts limit device performance, as in the presented study, the electronic model gained from KFM scans under different bias conditions sheds light on the electronic transport even at individual contacts. In the absence of a source–drain bias, and by controlling the carrier density in the nanowire through a gate electrode, KFM measurements also provide access to the density of surface states and sub-bands, as demonstrated before for InAs [28–29]. Characterization of three-terminal devices in terms of output and transfer characteristics, together with spatially resolved measurements of the electrostatic landscape by KFM, enable one to obtain complete and detailed electronic device models.

Additional SThM measurements make it possible to build the complementary thermal model of the device. For our nanowire device, the thermal coupling to the substrate and electrodes turn out to be of similar magnitude, and heat produced at the contacts can thus diffuse into the nanowire. This explained the increased temperature near the electrodes and the unusual asymmetric inverse parabolic shape of the thermal measurements. While similar thermal profiles had been observed before for 120 nm InAs nanowires [12], the origin of asymmetry and shape could not be clarified with certainty before.

Combined KFM and SThM will also prove useful to study nanowire devices with additional interfaces, e.g., due to doping gradients. The nanoscale lateral resolution of both methods makes it possible to investigate electronic and thermal properties of individual junctions. Combined experiments on silicon nanowires have been attempted before, but the results and analysis remained qualitative [30]. Here, we have shown that experimental KFM and SThM methods in fact can be used to obtain quantitative models, if parasitic effects, such as topography crosstalk and long-range averaging in KFM [9,20], or the varying thermal probe–surface resistance [12], are carefully accounted for, or excluded by the measurement technique.

## Experimental

### Sample preparation

Indium arsenide nanowire devices were fabricated on highly doped (ρ = 2 mΩ·cm) silicon wafers coated with a 100 nm thick thermal oxide. Electrical contact pads and alignment marks for e-beam alignment and localization of the NWs where defined and fabricated using an ultraviolet lithography process. The InAs NWs were transferred from a growth substrate onto the pre-patterned wafer on which they where localized and contacted by e-beam lithography. The InAs NWs studied were grown in a metal-organic vapor deposition (MOCVD) system by vapor–liquid–solid (VLS) growth using a gold particle as catalyst and using trimethylindium (TMIn) and *tert*-butylarsine (TBA) as precursors. The InAs NW studied has a diameter of 60 nm and was contacted by Au/Ni metal contacts of double the NW thickness. Prior to metal deposition, the native oxide shell around the InAs wire was removed by a dip in buffered hydrofluoric acid (BHF) giving rise to a slight tapering of the NW towards the contact region as visible in Figure S1 and Figure S2 (Supporting Information File 1).

### Scanning thermal microscopy

Our SThM setup relies on a cantilever with an integrated resistive heater, whose resistance is measured in a Wheatstone bridge configuration (Figure 1a). To be sensitive to changes of the resistance, the bridge voltage is measured with a differential amplifier. The heater voltage, *U*_dc_, resulting from the bridge bias, *U*_tot_, is measured separately. The microscope was operated under vacuum conditions to prevent parasitic heat paths between heater/sensor and sample surface. Sample temperature fields are reconstructed from the detected *U*_dc_ and *U*_ac_ raw voltage signals [21].

### Kelvin probe force microscopy

KFM measurements are performed in air using a commercial AFM (Cypher, Asylum Research) and an external lock-in amplifier (HF2, Zurich Instruments). Topography is acquired with net-attractive interactions in amplitude-modulation mode (*A*_free_ = 8.5 nm, *A*_set_ = 7.7 nm) using an Olympus AC160TS-R3 cantilever (*f*_0_ = 323.2 kHz, *k* = 40 N·m^−1^, *Q* = 500). To obtain the surface potential simultaneously with topography, we modulate the voltage applied to the tip (*U*_ac_ = 2 V at *f*_m_ = 4 kHz) on top of a dc bias, and we detect modulations of the force gradient from the sidebands of the drive frequency *f*_d_ in the deflection signal. The sidebands at *f*_d_ ± *f*_m_ are minimized by matching the dc tip bias to *U*_lcpd_ using a feedback loop. The sidebands at *f*_d_ ± 2*f*_m_ are proportional to the tip–sample capacitance gradient *C*′′ and the KFM sensitivity. The feedback loop in our setup uses both pairs of sidebands and a Kalman filter to continuously estimate the surface potential and to avoid topographical artefacts [20].

## Supporting Information

File 1 Additional SEM and AFM measurements, a description of the resistivity reconstruction method, derivation of the transmission line method, and details about the simulation and fit of temperature profiles.
