# Supplementary material for: Combined scanning probe electronic and thermal characterization of an indium arsenide nanowire
Source: Beilstein J Nanotechnol. 2018 Jan 11;9:129–36. doi: 10.3762/bjnano.9.15 (PMC5789438; doi:10.3762/bjnano.9.15)
Supplement: File 1 — Additional SEM and AFM measurements, a description of the resistivity reconstruction method, derivation of the transmission line method, and details about the simulation and fit of temperature profiles. [file Beilstein_J_Nanotechnol-09-129-s001.pdf]

Supporting Information

for

Combined scanning probe electronic and thermal characterization of an indium arsenide nanowire

Tino Wagner*[1], Fabian Menges[2], Heike Riel[2], Bernd Gotsmann[2] and Andreas Stemmer*[1]

Address: [1]ETH Zürich, Nanotechnology Group, Säumerstrasse 4, 8803 Rüschlikon, Switzerland and [2]IBM Research – Zurich, Säumerstrasse 4, 8803 Rüschlikon, Switzerland

Email: Tino Wagner* - tiwagner@ethz.ch, Andreas Stemmer* - astemmer@ethz.ch

* Corresponding author

1 Complementary SEM and AFM measurements

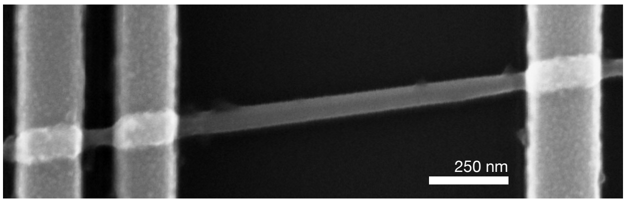

Figure S1: SEM micrograph of the 60 nm InAs nanowire, contacted with Au electrodes (bright) on a Si/SiO_2 substrate (dark).

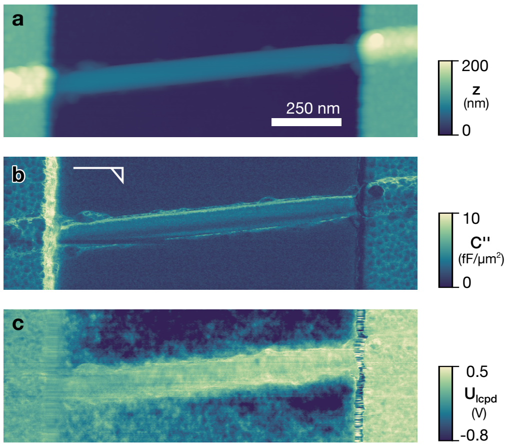

Figure S2: KFM scan of the nanowire. Topography (a), capacitance gradient C'' (b), and surface potential (c) of the scan shown in fig. 2(b). Note that the increased capacitance gradient at the edge of the left electrode is due to an increased interaction with the AFM tip (inset).

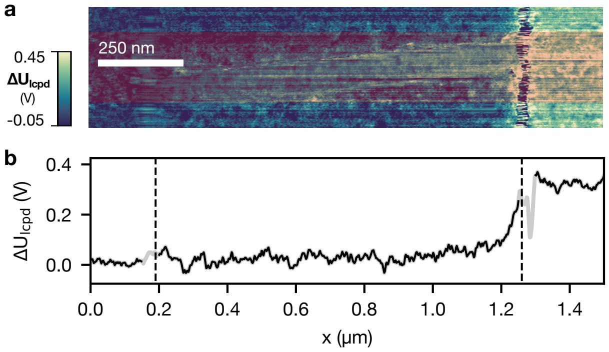

Figure S3: Difference of biased and grounded surface potential next to the nanowire. (a) KFM image at zero bias subtracted from a scan biased at 37.7 µA. (b) Column average of (a), excluding the area containing the nanowire (shaded red). Dashed lines indicate outer edges of the electrodes. In the oxide-covered areas, the influence of the bias applied to the right electrode can only be seen in a narrow region closest to the electrode edge. This could be due to a remaining stray capacitive coupling of the AFM tip to the 120-nm high electrode, or it could be an effect of populating trap states in the oxide.

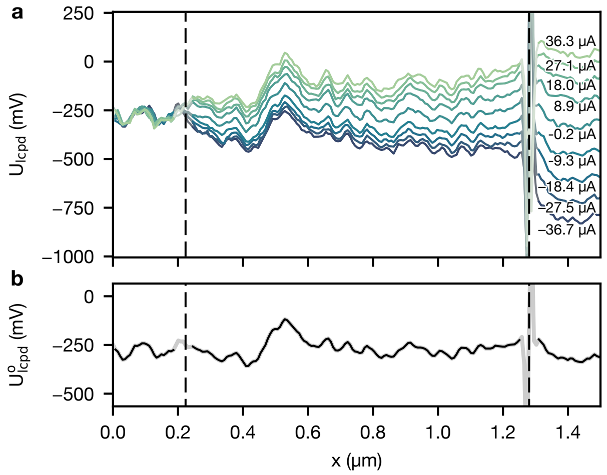

Figure S4: KFM raw data during sweeps of the source–drain current. (a) Measured raw surface potential profiles. (b) Bias-independent offset extracted from (a) using the reconstruction algorithm.

2 Reconstruction of wire resistivity and contact resistances

The algorithm determines a viable arrangement of (non-linear) resistors such that the resulting voltage profiles under current bias match the line profiles measured by KFM. This arrangement contains information about the wire resistivity and contact resistances.

The resistors form a chain as follows, where $\circ$ indicates a node at fixed potential U, and R_i and R_c^ν are the chain and contact resistances, respectively:

$\circ(U_{el}^l) — R_c^l — \circ — R_1 — \circ — R_2 — \circ — \ldots — \circ — R_n — \circ — R_c^r — \circ(U_{el}^r)$

We may assume that one electrode is held at ground potential, $U_{el,l} = 0$. As a function of position, the potentials are

$$U(x < x_{el}^l) = 0 \tag{1}$$

$$U(x_{el}^l) = R_c^l(I)I \tag{2}$$

$$U(x_{el}^l \leq x \leq x_{el}^r) = U(x_{el}^l) + \int_{x_{el}^l}^{x} \mathrm{d}x\, \rho'(x,I)I \tag{3}$$

$$U(x_{el}^r \leq x) = U(x_{el}^r) + R_c^r(I)I \tag{4}$$

where $\rho'(x) = \rho/A$ is the distributed wire resistance, or wire resistivity.

The full profile is given by the contact resistance and the wire resitivities. For the calculation, the integral is discretized using the trapezoidal rule and $\rho(x_i,I) = R_i(I)/\Delta x$.

$R_c^{l,r}$ and ρ' are assumed to follow a power series of the current I, i.e.

$$\rho'(x,I) = \alpha(x) + \beta(x)I + \gamma(x)I^2 + \cdots + \nu(x)I^n$$

for terms up to n-th order.

The reconstruction algorithm minimizes the functional

$$\frac{1}{2}\sum_I \left(U(x,I) + U_{\text{offset}}(x) - U_{\text{lcpd}}(x,I)\right)^2 \tag{5}$$

$$+ \quad \tau\sum_I \left|\partial\rho'(x,I)/\partial x\right|_1 \tag{6}$$

$$+ \quad \nu\sum_I \left|\partial^2\rho'(x,I)/\partial x^2\right|_1 \tag{7}$$

The last two terms are the total variation of $\rho'(x,I)$ and $\partial\rho'(x,I)/\partial x$. Thereby, we can balance the total variation of ρ' and its derivative with the least-squares search of an optimal voltage offset $U_{\text{offset}}(x)$ and wire profile $U(x,I)$. Essentially, τ and ν govern the smoothness of the resulting fit of the resistivity and its derivative, respectively, by balancing the least-squares functional, eq. (5), with terms penalizing spatial variations of $\rho'(x,I)$ and $\partial\rho'(x,I)/\partial x$. The ℓ_1 norm biases the search towards sparse solutions; no change at all is considered better than minor changes of ρ'.

In addition to the functional above, the solution can be constrained by the following conditions:

- Symmetry of $\rho'(x,I)$ and $R_c^{l,r}$ around $I = 0$
- Continuity of $\rho'(x,I)$ and $R_c^{l,r}$ at $I = 0$
- Positivity of ρ' and $R_c^{l,r}$
- Equality of $U_{\text{offset}}(x)$ at both electrodes, except for metals with dissimilar work function.

The optimization is performed using CVXPY [1] and the ECOS [2] solver. The regularization parameters ($\tau = 25$, $\nu = 1000$) are chosen to avoid overfitting of the data (resampled to 212 points, $\Delta x = 7.4\,\mathrm{nm}$), and to obtain a smooth resistivity profile.

3 Model of a leaky diode

The current–voltage characteristic of an idealized diode is given by the Shockley diode equation

$$I_\mathrm{d}(U, I_\mathrm{s}, T, \eta) = I_\mathrm{s}\left(\exp\left(qU/\eta kT\right) - 1\right), \tag{8}$$

where I_s is the saturation current, U is the applied voltage, q the elementary charge, k the Boltzmann constant, and T the temperature. η is an ideality factor, which is close to unity for thermionic emission [3]. Adding a resistance R_p in parallel to this diode, the current through a leaky diode is:

$$I = I_\mathrm{d}(U, I_\mathrm{s}, T, \eta) + U/R_\mathrm{p}. \tag{9}$$

4 Transmission line model for contact resistances

We assume a transmission line model for current injection at the electrodes [4-6]. The model describes the wire as a chain of linear, infinitesimal resistors. Each element, having a resistance $R'\mathrm{d}x$ (distributed resistance $R' = \mathrm{d}R/\mathrm{d}x =: \rho'$), is connected to the electrode via the conductance $G'\mathrm{d}x$ (distributed conductance G'). For a grounded electrode, the following system of equations describes the behaviour at the contact,

$$U'(x) + R'I(x) = 0 \tag{10}$$

$$I'(x) + G'U(x) = 0, \tag{11}$$

where $I(x)$ and $U(x)$ are the current and voltage of the wire segment at x, respectively. R' is related to the bulk resistivity ρ (in $\Omega\,\mathrm{m}$) of the wire by its cross-section A, and G' is related to the contact

width W and the contact resistivity ρ_c (in $\Omega\,\mathrm{m}^2$) via

$$R' = \frac{\rho}{A} \quad \text{and} \quad G' = \frac{W}{\rho_c}. \tag{12}$$

Equations (10) and (11) are solved for $x \geq 0$ by

$$U(x) = U_+ \exp(-\gamma x) + U_- \exp(\gamma x) \tag{13}$$

$$I(x) = \frac{1}{R_0} \left[U_+ \exp(-\gamma x) - U_- \exp(\gamma x) \right] \tag{14}$$

$$L_\mathrm{T} = \frac{1}{\gamma} = \frac{1}{\sqrt{R'G'}} = \sqrt{\frac{\rho_c A}{\rho W}} \tag{15}$$

$$R_0 = R' L_\mathrm{T} = \frac{1}{G' L_\mathrm{T}}. \tag{16}$$

where L_T is the transfer length. U_+ and U_- depend on the exact boundary conditions, i.e., the contact length L and the line termination. If the line is terminated at L with R_L, then $U(L) = R_\mathrm{L} I(L)$. Using eqs. (13) and (14) we find

$$r := \frac{U_- \exp(\gamma L)}{U_+ \exp(-\gamma L)} = \frac{R_\mathrm{L} - R_0}{R_\mathrm{L} + R_0}, \tag{17}$$

with the reflection coefficient r, which balances U_+ and U_- given the boundary conditions. For termination with infinite resistance ($R_\mathrm{L} \to \infty$, $r \to 1$), we obtain the following well-known expressions for $U(x)$, $I(x)$, and the contact resistance R_c [5]

$$U(x) = R_0 I_0 \frac{\cosh\left((L-x)/L_\mathrm{T}\right)}{\sinh\left(L/L_\mathrm{T}\right)} \quad \text{and} \quad I(x) = I_0 \frac{\sinh\left((L-x)/L_\mathrm{T}\right)}{\sinh\left(L/L_\mathrm{T}\right)} \tag{18}$$

$$R_c := \frac{U(0)}{I(0)} = R_0 \coth\left(\frac{L}{L_\mathrm{T}}\right) = R' L_\mathrm{T} \coth\left(\frac{L}{L_\mathrm{T}}\right). \tag{19}$$

Because of the top-contact electrodes in the experiment, the wire potential $U(x)$ is inaccessible below the electrodes. With R_c and R' known from the KFM measurement and analysis, the transfer length L_T can be estimated from a numerical solution of eq. (19). Once L_T is known, the contact

resistivity $\rho_c = R'WL_T^2$ can be determined. In Figure S5 we show L_T and ρ_c as a function of bias current, where the wire resistivity $R' = \rho'$ and contact resistance R_c are taken from fig. 3 of the main text.

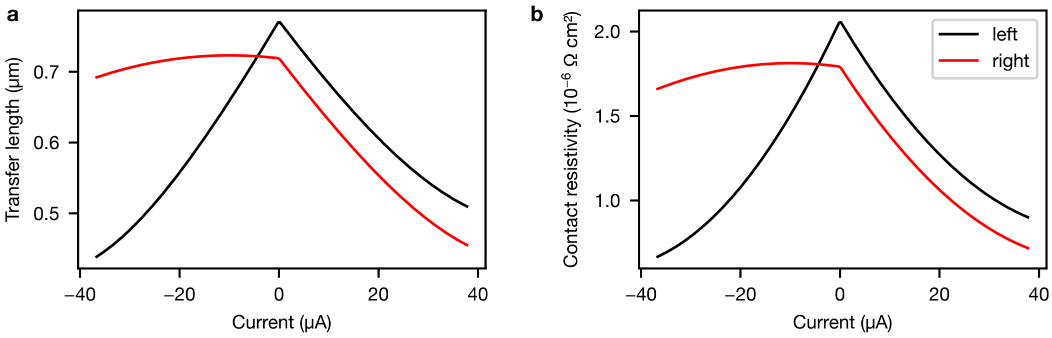

Figure S5: Transfer lengths and contact resistivities of the two contacts. Transfer length (a) and contact resistivity (b) as a function of bias current. (Wire resistivity $\rho' = 3.7\,\mathrm{k\Omega\,\mu m^{-1}}$; contact lengths $L_{\mathrm{left}} \approx 420\,\mathrm{nm}$, $L_{\mathrm{right}} = 250\,\mathrm{nm}$; contact width $W = \pi d_{\mathrm{wire}}/2 = 94\,\mathrm{nm}$)

The current injected into the wire at x is found from $I_{\mathrm{inj}}(x) = -I'(x) = G'U(x)$. For long contacts $L \gg L_T$, both $I(x)$ and $I_{\mathrm{inj}}(x)$ share the same exponential decay $\propto x/L_T$, since $U_- \to 0$. For short contacts, this is no longer the case and the expressions in eq. (18) must be used.

We find the total power dissipated, $P'_{\mathrm{tot}}(x)$, as the sum of the power through the contact, $P'_{\mathrm{inj}}(x) = G'U^2(x)$, and the power dissipated in the wire, $P'(x) = R'I^2(x)$:

$$P'_{\mathrm{tot}}(x) = P'(x) + P'_{\mathrm{inj}}(x) = R'I^2(x) + G'U^2(x) \tag{20}$$

$$= \frac{R_0 I_0^2}{L_T} \frac{\cosh\left(2(L-x)/L_T\right)}{\sinh^2\left(L/L_T\right)} \tag{21}$$

Integrated over the full length, we obtain the total power dissipated at the contact,

$$\int_0^L \mathrm{d}x\, P'_{\mathrm{tot}}(x) = R_c I_0^2. \tag{22}$$

5 Simulation and fit of temperature profiles

5.1 Heat equation

We now derive the one-dimensional heat equation for a self-heated nanowire coupled to a substrate (and electrodes). As sketched in Figure S6, the infinitesimal wire segment of length dx at the position x, has the cross-section A and is in contact with the substrate over the width W.

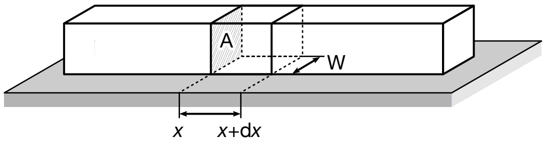

Figure S6: Schematic view of the nanowire on a substrate, showing the cross-section A and thermal contact width W.

A temperature change of this element is related to a change of its internal energy Q_{tot} [7],

$$\dot{Q}_{\text{tot}} = c_p \rho A \frac{\partial T}{\partial t} \mathrm{d}x, \tag{23}$$

where ρ and c_p are the density and specific heat capacity of the nanowire, respectively, and T is the temperature. The energy change $\dot{Q}_{\text{tot}}$ must equal the heat flux into and out of the element, $\dot{Q}_{\text{in}}$ and $\dot{Q}_{\text{out}}$, heat loss to the substrate $\dot{Q}_{\text{loss}}$, and Joule heating of the element Q_{j}, each with their respective sign:

$$\dot{Q}_{\text{tot}} = -(\dot{Q}_{\text{out}} - \dot{Q}_{\text{in}}) - \dot{Q}_{\text{loss}} + \dot{Q}_{\text{j}}. \tag{24}$$

By Fourier's law, the heat flow into and out of the element are [7]

$$\dot{Q}_{\text{in}} = -\kappa A \left.\frac{\partial T}{\partial x}\right|_{x} \quad \text{and} \quad \dot{Q}_{\text{out}} = -\kappa A \left.\frac{\partial T}{\partial x}\right|_{x+\mathrm{d}x}, \tag{25}$$

where κ is the thermal conductivity of the nanowire. In the limit of $\mathrm{d}x \to 0$, we find

$$-(\dot{Q}_{\mathrm{out}} - \dot{Q}_{\mathrm{in}}) = \frac{\partial}{\partial x}\left[\kappa A \frac{\partial T}{\partial x}\right] \mathrm{d}x. \tag{26}$$

Similarly, heat loss to the substrate is governed by heat conduction (κ_{s}) through the cross-section $W\mathrm{d}x$ with the substrate (temperature T_0) and a small separation t_{s},

$$\dot{Q}_{\mathrm{loss}} = -\kappa_{\mathrm{s}} W \frac{T_0 - T}{t_{\mathrm{s}}} \mathrm{d}x = \tau W (T - T_0)\, \mathrm{d}x, \tag{27}$$

where we have introduced the thermal conductivity to the substrate, $\tau := \kappa_{\mathrm{s}}/t_{\mathrm{s}}$ (in $\mathrm{W\,m^{-2}\,K^{-1}}$). The product of τ and W defines the thermal conductance to the substrate per unit length (in $\mathrm{W\,m^{-1}\,K^{-1}}$), $g := \tau W$. Note that eq. (27) assumes that the substrate next to the nanowire is at the same temperature T_0 everywhere, i.e. the substrate spreading resistance is negligible compared to the interfacial resistance.

Joule heating for the element at x is

$$\dot{Q}_{\mathrm{j}} = \dot{q}_{\mathrm{j}} \mathrm{d}x, \tag{28}$$

where $\dot{q}_{\mathrm{j}} = \rho'(x) I^2$ is the power dissipated per unit length for a current I driven through the wire (linear resistivity ρ').

Combining eqs. (23) to (28), we obtain the one-dimensional heat equation for a self-heated nanowire, also given by [8],

$$c_p \rho A \frac{\partial T}{\partial t} = \frac{\partial}{\partial x}\left[\kappa A \frac{\partial T}{\partial x}\right] - \tau W (T - T_0) + \dot{q}_{\mathrm{j}}. \tag{29}$$

In the steady-state, $\partial T / \partial t \to 0$, we find

$$-\frac{\partial}{\partial x}\left[\kappa A \frac{\partial T}{\partial x}\right] + \tau W (T - T_0) = \dot{q}_{\mathrm{j}}. \tag{30}$$

We posed no restriction on the nature of κ, τ, A, and W, i.e., they may also be functions of x.

For the solution, we assume Dirichlet boundary conditions

$$T(x=0) = T_0 \quad \text{and} \quad T(x=L) = T_0, \tag{31}$$

where L is the total wire length.

5.2 Numerical solution

We only consider the steady-state heat equation here, eq. (30), i.e., we assume that for each bias applied to the nanowire device, the temperature field follows immediately. This is a reasonable assumption when the time constant $\tau = A/\alpha$ determined by the thermal diffusivity $\alpha = \kappa/c_\mathrm{p}\rho$ is negligible.

Furthermore, we assume uniform thermal conductance κ along the nanowire with a uniform cross-section $A = \pi(60\,\mathrm{nm}/2)^2$. We assume the same thermal conductance $g_\mathrm{e} = \tau_\mathrm{e} W_\mathrm{e}$ of the nanowire segments to the left and right electrodes. Similarly, we choose the same conductance to the substrate $g_\mathrm{s} = \tau_\mathrm{s} W_\mathrm{s}$ for the center region between the electrodes.

For *dc* operation, heat generated in the nanowire is due to Joule heating, $\dot{q}_\mathrm{j} = \rho'(x) I^2$. For heat dissipated at the contacts, we consider the power distribution obtained from the transmission line model, eq. (21).

To compare with temperature profiles obtained by SThM under *ac* bias, we calculate for each *ac* current the average power dissipated per unit length in the wire and along the contacts. This is done by calculating the instantaneous power during a sinusoidal current bias, and finally integrating over one period. At the contacts, we average in this process over the different contact resistivities and transfer lengths.

For the numerical solution, eq. (30) is discretized with central differences, and the resulting linear system is solved numerically for the temperature.

5.3 Fit and error estimation

To obtain κ, g_s, and g_e, we perform a least-squares fit of the calculated temperature field $\hat{T}$ (via eq. (30)) to the profiles T obtained by SThM for each power P,

$$\{\kappa, U_\mathrm{s}, U_\mathrm{e}\} = \operatorname{argmin} \sum_{P_j \in \mathbf{P}} \sum_{x_i \in \mathbf{x}} \frac{1}{\sigma_\mathrm{T}^2(x_i, P_j)} \left(\hat{T}(x_i, P_j, \kappa, U_\mathrm{s}, U_\mathrm{e}) - T(x_i, P_j)\right)^2, \tag{32}$$

where $\sigma_T(x_i, P_j)$ describes the temperature uncertainty of the measurements. The fit is performed using the `optimize.least_squares` method from SciPy [9]. Errors in the fit parameters are calculated from the diagonal elements of the fit covariance matrix.

The fit requires proper alignment of SThM and KFM profiles, which is not straightforward due to different tip convolution with the SThM and KFM probes in topography. For this reason, we only consider the center wire segment in the fit and exclude contact edges. To estimate the worst-case temperature error due to data alignment, we calculate the maximum squared deviation from shifting the temperature data $T(x)$ by a distance δ. For discrete temperature samples $y_i = T(x_i)$ with $x_i = i\Delta x$, and $h \equiv \delta/\Delta x$, we have

$$\sigma_\mathrm{max}^2(h) = \max_{i \in [1, N-h]} \left\{ (y_{i+h} - y_i)^2 \right\}. \tag{33}$$

The maximum squared deviation resulting from the alignment uncertainty σ_x is obtained by weighting with a Gaussian window ($\propto \exp(-(h\Delta x)^2/2\sigma_x^2)$) integrating eq. (33). With $\sigma_\mathrm{x} = 10\,\mathrm{nm}$, the worst-case error for the dataset in fig. 1 of the main manuscript is $\sigma_\mathrm{T,shift} = 0.23\,\mathrm{K}$, whereas the measurement noise level amounts only to $\sigma_\mathrm{T,meas} = 31\,\mathrm{mK}$. The total uncertainty in temperature is calculated from

$$\sigma_\mathrm{T}^2(x_i, P_i) = \sigma_\mathrm{T,meas}^2 + \sigma_\mathrm{T,shift}^2. \tag{34}$$

Figure S7 shows the best-fit parameter values with 2σ error bars.

We estimate the relative accuracy of the temperature calibration in our SThM measurements to

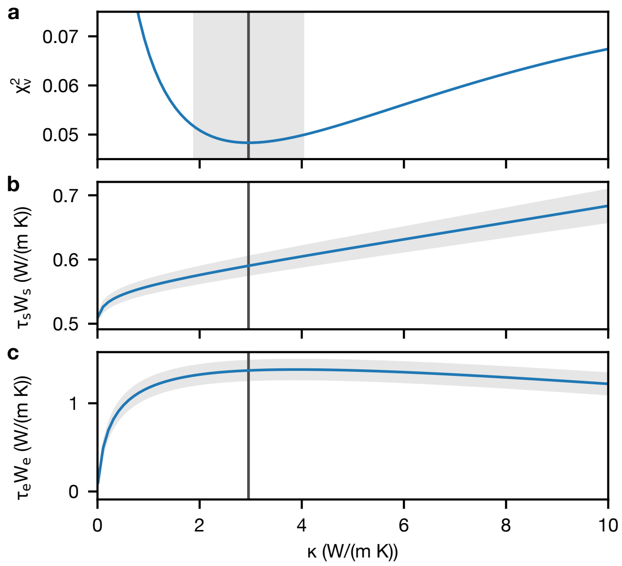

Figure S7: Substrate and electrode couplings fitted as a function of the nanowire thermal conductivity κ. (a) Mean square weighted deviation of the fit from scanning thermal measurements, $\chi^2_\nu = \sum(\hat{T} - T)^2/(\nu\sigma^2_\mathrm{T})$, with ν degrees of freedom. $\chi^2_\nu \ll 1$ indicates a good fit within the specified errors. (b) Thermal conductance to substrate $g_\mathrm{s} = \tau_\mathrm{s} W_\mathrm{s}$. (c) Thermal conductance to electrodes $g_\mathrm{e} = \tau_\mathrm{e} W_\mathrm{e}$. Error bars ($2\sigma$) are calculated given the total uncertainty of thermal measurements, which includes a worst-case estimate of the error resulting from the alignment ($\sigma_x = 10\,\mathrm{nm}$) of experimental data with the nanowire model. To account for the accuracy of the temperature calibration, a relative error of 30 % must be considered in addition.

$\eta \approx 25\,\%$ [10] with respect to the substrate temperature T_0. From the heat equation in eq. (30), it follows that after a change of the temperature to $(1 \pm \eta)(T - T_0)$, both the thermal conductivity κ and the thermal interface conductivity τ must change by a factor of $1/(1 \pm \eta)$. Therefore, we estimate the systematic relative error due to temperature calibration to

$$\left|\frac{\kappa' - \kappa}{\kappa}\right| = \left|\frac{\tau' - \tau}{\tau}\right| = \left|\frac{1}{1 \pm \eta} - 1\right| \approx \left|-\eta + \eta^2\right| < \eta + \eta^2 \approx 30\,\%. \tag{35}$$

The errors stated in the main text include both the random error, due to eq. (34), and systematic error, eq. (35).

References

1. Diamond, S.; Boyd, S. *Journal of Machine Learning Research* **2016**, *17* (83), 1–5.

2. Domahidi, A.; Chu, E.; Boyd, S. ECOS: An SOCP solver for embedded systems. In *European Control Conference (ECC)*; 2013; pp 3071–3076.

3. Sze, S. M.; Ng, K. K. *Physics of Semiconductor Devices*, 3rd ed.; Wiley-Interscience, 2006.

4. Shockley, W. *Research and investigation of inverse epitaxial UHF power transistors, Technical Report No. AL TDR 64-207*; AF Avionics Laboratory, 1964.

5. Berger, H. H. *Solid-State Electron.* **1972**, *15* (2), 145–158.

6. Berger, H. H. *J. Electrochem. Soc.* **1972**, *119* (4), 507–514.

7. Lienhard IV, J. H.; Lienhard V, J. H. *A Heat Transfer Textbook*, 4th ed.; Phlogiston Press: Cambridge, MA, 2016.

8. Shi, L.; Zhou, J.; Kim, P.; Bachtold, A.; Majumdar, A.; Mceuen, P. L. *J. Appl. Phys.* **2009**, *105* (10), 104306.

9. Jones, E.; Oliphant, T.; Peterson, P. et al. SciPy: Open source scientific tools for Python. http://www.scipy.org/ (accessed July 12, 2017).

10. Menges, F.; Riel, H.; Stemmer, A.; Gotsmann, B. *Nano Lett.* **2012**, *12* (2), 596–601.
